# Use of Organic Acid Mixtures Containing 2-Hydroxy-4-(Methylthio) Butanoic Acid (HMTBa) to Mitigate *Salmonella enterica*, Shiga Toxin-Producing *Escherichia coli* (STEC) and *Aspergillus flavus* in Pet Food Kibbles

**DOI:** 10.3390/ani13050877

**Published:** 2023-02-28

**Authors:** Aiswariya Deliephan, Janak Dhakal, Bhadriraju Subramanyam, Charles G. Aldrich

**Affiliations:** 1 Department of Grain Science and Industry, Kansas State University, Manhattan, KS 66506-2201, USA; 2 Department of Food Science & Technology, University of Nebraska-Lincoln, Lincoln, NE 68588-6205, USA

**Keywords:** pet food, kibble, antimicrobial, HMTBa, *Salmonella*, *Escherichia coli*, STEC, *Aspergillus*

## Abstract

**Simple Summary:**

Pathogens such as bacteria and molds have been contaminating foods including pet foods over the years. Contaminated pet food, apart from making pet animals sick, can also cause food-borne illnesses in humans as they are often handled by pet owners and children at home. In this study, we evaluated two types of organic acid mixtures which when applied as coating on pet food kibbles, could potentially mitigate pathogenic bacteria and molds during post-processing operations in the pet food industry. Our results indicated that the organic acid mixtures were considerably effective in mitigating the growth of the targeted pathogens in kibbles. These organic acid mixtures also have an additional advantage of being food-safe ingredients that are safe for consumption for the animals, and also provide some gut health benefits for them.

**Abstract:**

Post-processing operations of extruded pet food kibbles involve coating the product with fats and flavorings. These processes increase the risk for cross-contamination with food-borne pathogens such as *Salmonella* and Shiga toxin-producing *Escherichia coli* (STEC)*,* and mycotoxin-producing molds such as *Aspergillus* spp. after the thermal kill step. In this study, the antimicrobial effects of two types of organic acid mixtures containing 2-hydroxy-4-(methylthio) butanoic acid (HMTBa), Activate DA™ and Activate US WD-MAX™, against *Salmonella enterica,* STEC and *Aspergillus flavus* when used as a coating on pet food kibbles were evaluated. Using canola oil and dry dog digest as fat and flavor coatings, the efficacy of Activate DA (HMTBa + fumaric acid + benzoic acid) at 0%, 1% and 2%, and Activate US WD-MAX (HMTBa + lactic acid + phosphoric acid) at 0%, 0.5% and 1% was tested on kibbles inoculated with a cocktail of *S. enterica* serovars (Enteritidis, Heidelberg and Typhimurium) or Shiga toxin-producing *E. coli* (STEC) serovars (O121, and O26) at 37 °C for 0, 12, 24, 48, 72 h, 30 and 60 days. Similarly, their efficacy was tested against *A. flavus* at 25 °C for 0, 3, 7, 14, 21, 28 and 35 days. Activate DA at 2% and Activate US WD-MAX at 1% reduced *Salmonella* counts by ~3 logs after 12 h and 4–4.6 logs after 24 h. Similarly, STEC counts were reduced by ~2 logs and 3 logs after 12 h and 24 h, respectively. Levels of *A. flavus* did not vary up to 7 days, and afterwards started to decline by >2 logs in 14 days, and up to 3.8-log reduction in 28 days for Activate DA and Activate US WD-MAX at 2% and 1%, respectively. The results suggest that the use of these organic acid mixtures containing HMTBa during kibble coating may mitigate post-processing enteric pathogen and mold contamination in pet food kibbles, with Activate US WD-MAX being effective at a lower concentration (0.5–1%) compared to Activate DA.

## 1. Introduction

A large percentage of pet owners prefer feeding dry commercial pet foods to their pets due to the convenience and nutritional benefits. Dry pet food constitutes the most commonly sold type of pet food in the world, forming 75.2% of dog food and 53.9% of cat food categories [1]. These foods contain a variety of plant and animal ingredients as raw materials. The animal proteins are processed by different methods, predominantly in rendering plants, where there is a risk of contamination with various pathogens such as *Salmonella* [2]. The gastrointestinal tract of pets that consume these contaminated foods may get colonized with these bacteria, and yet they may not show any clinical symptoms [3]. These occurrences confirm that pathogens such as *Salmonella* can be transmitted to humans through the handling of dry pet foods [4]. Numerous cases of human salmonellosis have been linked to contaminated dry pet food such as dog and cat food kibbles [3], and animal feed [5]. Although extruded pet food like kibbles go through a thermal kill step during processing, they can become cross-contaminated with pathogenic bacteria during subsequent processing steps such as coating with fats and flavors [6]. By handling or consuming these foods, they may get transmitted to the pet which can serve as a carrier to humans. For instance, from 2006 to 2008, an outbreak of *Salmonella enterica* serotype Schwarzengrund, included 79 illnesses over 21 states, resulted in the recall of 105 brands of dry pet food and permanently closed a manufacturing plant in Pennsylvania [7]. The process validation in the facility included a specified time-temperature combination to kill *Salmonella*, and then the food was subsequently moved to coating and flavoring steps where it was sprayed with fat and palatants. An epidemiological investigation led to the isolation of the bacterial strain related to the outbreak from the flavoring room, meaning that the *Salmonella* contamination occurred during that step [8]. In 2012, there was also an outbreak of human *Salmonella enterica* serotype Infantis infections related to exposure to dry dog food [9].

*Escherichia coli* are some of the most prevalent enteric bacteria in animals and humans, and are also important zoonotic agents, which can be implicated in animal and human infectious diseases [10]. A recent study showed that antibiotic-resistant *E. coli* could easily spread between humans and their pets [11]. Shiga toxin-producing strains of *E. coli* O157:H7 have also caused numerous deaths following the consumption of contaminated foods [12]. Many studies on the prevalence of antimicrobial resistance in *E. coli* isolates from farm animals and pets have been performed in other countries beyond the United States [10,13,14]. In 2016, the U.S. Food and Drug Administration (FDA) along with the Centers for Disease Control and Prevention (CDC) investigated a multi-state outbreak of Shiga toxin-producing *E. coli* (STEC) O121 and O26 infections. Sixty-three people infected with the outbreak strains of STEC O121 or O26 were reported from 24 states, and it was traced back to contaminated wheat flour from a General Mills facility in Kansas City, MO. Although pet food recalls due to STEC contamination have not been reported yet, recalls involving STEC-contaminated wheat flours have increased compared to previous years with 13 recalls occurring in 2019. Wheat flour is commonly used as an ingredient in pet foods, so the potential for STEC contamination exists. Previous research studies have investigated physical (e.g., vacuum steam) and chemical (e.g., sodium bisulfate) intervention strategies in wheat flour milling to mitigate STEC contamination [15,16,17].

Another risk factor for human and animal food safety is the presence of fungi and the potential for mycotoxins that they may produce. Hazardous mycotoxins can occur in cereal grains due to stress during toxigenic fungal growth, can be compounded with improper storage and the process of cooking does not reduce their content [18]. During pet food manufacturing, the foods can be contaminated with mold spores especially when cereal grains are ground, and the foods are pelleted or formed [19]. Pet food kibbles with water activity of 0.50 are not very conducive for mold growth; however, mold spores prevalent in the environment can contaminate packaged foods that are opened by the consumer and can amplify during food storage, especially in a humid environment with >75% RH. Moldy foods reduce the nutritional value due to spoilage and under certain conditions may produce toxic metabolites called mycotoxins. These toxins with chemically diverse structures have been involved in disease outbreaks which have affected both animal and human health [20,21]. A study by Beuno et al. [22] identified commonly occurring molds in 21 pet foods including dog food kibbles across eight commercial brands produced in Argentina, and were comprised mainly of *Aspergillus*, followed by *Rhizopus* and *Mucor* spp. Fungal contamination can lead to economic losses associated with nutrient and palatability reduction. Furthermore, the presence of mycotoxins also affects both the animal’s and human’s health [23]. *Aspergillus flavus*, being the most reported in pet food, is responsible for the production of aflatoxins. Dogs are extremely sensitive to this group of toxins, with the liver being their main target [24]. In the U.S. in 2005, aflatoxicosis-related illnesses in dogs and *A. flavus*-contaminated dog food recalls were reported. More recently, in 2020, 28 deaths and eight illnesses were reported in dogs that consumed the recalled Sportmix™ pet food product that was contaminated with aflatoxin. Pet food recalls by Sunshine Mills also happened in 2020, due to aflatoxin contamination from corn that was used as an ingredient in the pet food [25]. Thus, there is a need to control or reduce toxigenic *A. flavus* contamination. 

**Figure 1:**
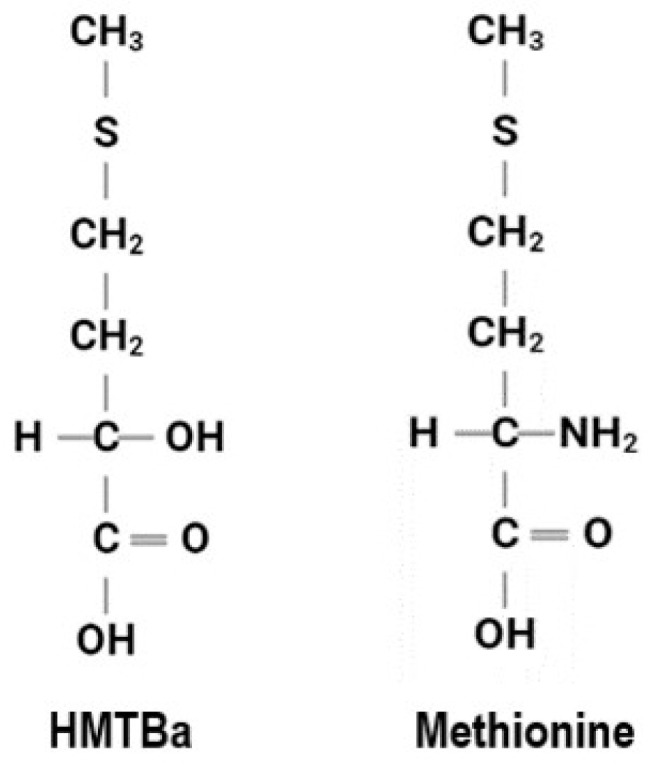
Comparison of chemical structures of HMTBa (methionine hydroxy analogue) and amino acid methionine.

The amino acid methionine is identified as a limiting amino acid in high forage cattle diets and has a positive impact on the health and performance of the animal. Methionine participates in a wide variety of metabolic pathways and serves as a precursor to other amino acids, such as cysteine. If free amino acids are fed directly to beef cattle, the rumen microbes destroy them before they even leave the rumen. Because amino acids must be presented to the small intestine in required amounts for the animal to synthesize protein, the amino acid must be protected or modified to avoid rumen degradation. Supplementing animal feed rations with a ‘methionine hydroxy analogue’ is an economical way to supply methionine. HMTBa (2-hyroxy-4-(methlythio) butanoic acid) is an organic acid and a methionine hydroxy analogue. It has been used as a methionine precursor in animal feed due to its unique chemical structure (Figure 1) that allows protection from some of the microbial degradation in the rumen gut. HMTBa also provides acidifying effects of organic acids. These acidifying effects subsequently provide gut health advantages to the animal by mitigating pathogen growth in the gut [26,27]. Methionine hydroxy analogue has also been shown to reduce nitrogen excretions [28], support animal performance during heat stress [29,30] and offer antioxidant capacity [31,32,33,34]. Other than these health benefits and its use as a methionine precursor in animal feed supplements, its potential role in enhancing food safety of processed foods or its application in pet foods has not been investigated so far. 

HMTBa is one of the main components of Activate DA™ and Activate US WD-MAX™, which are proprietary blends of organic acids from Novus International (St. Charles, MO, USA). According to the company’s product information, research conducted by the CCL Institute in the Netherlands using HMTBa demonstrated its effectiveness in reducing bacterial populations such as *Salmonella*, *E. coli* and *Campylobacter*, all of which can be found in the drinking water of poultry. The combination of organic acids in Activate DA and Activate US WD-MAX effectively reduces the pH of the gastrointestinal tract, promotes the establishment of a desirable and more balanced intestinal flora and aids in digestion, providing more nutrients from feed and improving the performance of the animal. Activate DA (HMTBa + fumaric acid + benzoic acid + silica + mineral oil) is a granular mixture applied to premixes and finished feeds. Activate US WD-MAX (HMTBa + lactic acid + phosphoric acid) is used for the acidification of drinking water for poultry, making the drinking water a less favorable environment for pathogen growth, and it is shown to play an important role in the destruction of harmful microorganisms in the gut that could affect the birds’ performance. Parker et al. [35] evaluated the organic acid mixture Activate WD™ containing HMTBa at 0.04% and 0.08% in drinking water for poultry and found a reduction in the horizontal transmission of *Salmonella* in the broiler chickens. Guo-zheng et al. [36] evaluated Activate WD against *Staphylococcus aureus*, *Escherichia coli*, *Salmonella pullorum* and *Campylobacter jejuni* in nutrient broth and found it to be effective at 0.6%. Deliephan [37] evaluated Activate DA and Activate US WD-MAX as sanitizers on food contact surfaces to inhibit *Salmonella*. Very few studies had evaluated chemical additive coating in pet food kibbles using organic acids like 3-hydroxy-3-methylbutyrate (HMB) [38] and medium chain fatty acids (caproic, caprylic, and capric) [6] to mitigate post-extrusion cross-contamination from *Salmonella*. There is limited knowledge on the application of organic acid mixtures containing HMTBa in dry pet food kibbles to enhance food safety.

The objectives of this research study were as follows: (i) to determine the efficacy of organic acid mixtures, Activate DA and Activate US WD-MAX, coated on extruded pet food kibble on the survival of *Salmonella enterica*, *Escherichia coli* (STEC) and *Aspergillus flavus*; and (ii) to evaluate the residual antimicrobial effect of the organic acid mixtures Activate DA and Activate US WD-MAX coated on extruded pet food kibble on *Salmonella enterica*, to maintain *Salmonella*-free kibble despite repeated exposure to recontamination over time.

## 2. Materials and Methods

### 2.1. Materials

Uncoated dry pet food kibbles were custom manufactured at Extru-Tech Inc. (Manhattan, KS, USA). The final moisture content of the kibble was 5.6% with water activity (a_w_) of 0.50. The composition of the kibble is presented in Table 1.

The organic acid mixtures evaluated in this study included Activate DA (dry formula) and Activate US WD-MAX (liquid formula) that were provided by the study sponsor Novus International (St. Charles, MO, USA). Activate DA was a mixture of HMTBa, fumaric acid, benzoic acid, silica and mineral oil. Activate US WD-MAX was a mixture of HMTBa, lactic acid and phosphoric acid. Activate DA, which was in granular form, was further ground to reduce its particle size using a laboratory hammer mill (Verder Scientific, Inc., Newtown, PA, USA) for use in this study.

### 2.2. Inoculum Preparation

*Salmonella enterica* serovars Enteritidis (ATCC 4931), Heidelberg (ATCC 8326) and Typhimurium (ATCC 14028) were procured from the American Type Culture Collection (ATCC, Manassas, VA, USA) and maintained in tryptic soy broth (TSB)-glycerol (7:3) at −80 °C. Before use, the frozen cultures were streaked onto tryptic soy agar (TSA; BD Difco, Sparks, MD, USA) plates and incubated at 37 °C for 24 h. A single colony of each *Salmonella* strain was inoculated into 10 mL of TSB (BD Difco, Sparks, MD, USA) and incubated at 37 °C for 18 to 24 h. The cultures of each *Salmonella* serotype thus obtained were centrifuged for 10 min at 5000× *g* (Thermo Scientific, Waltham, MA, USA) at room temperature. The pellets were resuspended in pre-sterilized 0.1% peptone water (Difco Laboratories, Sparks, MD, USA), and an equal volume of each serotype was mixed to obtain the cocktail (∼8 log CFU/mL).

Shiga toxin-producing *E. coli* (STEC) serovars O121 (ATCC 2219) and O26 (ATCC 2196) were maintained in tryptic soy broth (TSB)-glycerol (7:3) at −80 °C. The same procedure explained earlier for the preparation of the *Salmonella* cocktail was followed for the STEC cocktail preparation. The final STEC cocktail inoculum had a concentration of ∼8 log CFU/mL.

The fungal culture of aflatoxin-producing *Aspergillus flavus* (ATCC 15548) was maintained in potato dextrose broth (PDB)-glycerol (7:3) at −80 °C. Before use, the frozen cultures were streaked onto potato dextrose agar (PDA) with incubation at 25 °C for 72 h. The fungal spores were collected from the grown culture on PDA by adding 5 mL of 0.1% peptone water to the surface of the dish. The spores were then dislodged from the solid medium using an L-shaped plastic rod. The spore suspension in peptone water was then collected and stored at 4 °C and was used as the fungal inoculum (∼4 log CFU/mL).

### 2.3. MIC, MBC and MFC assays

The minimum inhibitory concentrations (MIC) of the organic acid mixtures were determined by the broth micro- and macro-dilution assay according to the antimicrobial susceptibility testing methods described by the Clinical and Laboratory Standards Institute [39]. To determine MIC, a 200 µL volume of Activate DA or Activate US WD-MAX consisting of twice the desired final concentration was dispensed in the first well of a 96-well microtiter plate (triplicate wells) and 100 µL of sterile water in the rest of the wells. A serial two-fold dilution of the organic acid mixtures was performed starting from 50% to 0.05% *v*/*v* concentration. A 100 μL aliquot of bacterial or fungal (*Salmonella* or STEC or *A. flavus*) culture (6 log CFU/mL for bacteria or 4 log CFU/mL for fungi) was added to each well of the plate already containing the 100 μL of decreasing concentrations of organic acid solutions to make a final volume of 200 μL per well. A positive control consisted of bacterial or fungal inoculum only (no treatment), and a negative control consisted of tryptic soy broth (TSB) or potato dextrose broth (PDB) only. The MIC was determined as the lowest concentration of organic acid mixture that inhibited visible growth of the target microorganism in the microtiter plate after 24 h (for bacteria) or 72 h (for fungi) of incubation at 37 °C or 28 °C, respectively. The inhibition of growth of the microorganisms was determined qualitatively by visual observation of turbidity in the growth media.

To determine minimum bactericidal concentration (MBC) and minimum fungicidal concentration (MFC), 100 µL of the sample from each well from the MIC experiment was plated onto xylose lysine deoxycholate (XLD) agar for *Salmonella*, potato dextrose agar (PDA) for *A. flavus* or 1 mL onto Petrifilm (3M, Minneapolis, MN, USA) plates for *E. coli* counts, for the enumeration of *Salmonella*, *A. flavus* and STEC colonies at each concentration of organic acid mixture after incubation at 37 °C for 24 h (for *Salmonella*) or 37 °C for 48 h (for STEC) or 28 °C for 72 h (for *A. flavus*). The colonies were enumerated manually. The MBC and MFC were determined as the lowest concentration of the organic acid mixture that caused an absence (≤1 colony) of bacterial or fungal growth on the plates or Petrifilm. The study was replicated three times.

### 2.4. Coating of Kibbles, Inoculation and Microbiological Analysis

To determine the antimicrobial efficacy of the organic acid mixtures, a 180 g aliquot of pet food kibbles for each treatment was transferred to a plastic container and autoclave sterilized. For each of the organic acid treatments (Activate US WD-MAX or Activate DA), for each pathogen tested (*Salmonella*, STEC or *A. flavus*), a total of six containers were maintained: four for each of the organic acid treatment concentrations (0.5% or 1% *w*/*w* of Activate US WD-MAX, and 1% or 2% *w*/*w* of Activate DA), one for the untreated and one for the negative control, for each pathogen tested. The organic acid mixtures were suspended in canola oil and applied to the kibbles using a pipette and mixing to coat them thoroughly to make a final weight of 200 g in each container. The final oil percentage on the kibbles was maintained at ~7–8%. The concentrations of the organic acid mixtures used were based on the MICs. The ‘untreated’ was without any organic acid treatment and inoculated with only the bacterial or fungal inoculum in TSB or PDB. The negative control was canola oil-coated kibble, without any organic acid treatment and without inoculation with bacteria or fungi. After 30 min of the organic acid treatment (coating), a bulk-harvested *Salmonella* culture or STEC culture cocktail (~8 log) was spot inoculated on the kibbles using a pipette. The initial moisture content of the kibbles was 5.6% and the final moisture content post-inoculation was maintained at ~8–9% dry basis. For example, for 0.5% Activate US WD-MAX treatment, 180 g kibbles + 14 g canola oil + 1 g organic acid (Activate US WD-MAX) + 5 mL inoculum was used to make a total weight of 200 g in the container. After uniform mixing of the kibbles, the containers were incubated at 37 °C. Similarly, for the fungal inoculation, *A. flavus* culture inoculum in PDB (~4 log) was spot inoculated on the kibbles. After uniform mixing of the kibbles, the containers were incubated at 28 °C. Microbiological analyses for *Salmonella* and STEC were conducted for each of the containers at various pre-determined time intervals: 2, 12, 24, 48, 72 h, 30 and 60 days, and the fungal analyses for *A. flavus* were conducted at 1, 3, 7, 14, 21, 28 and 35 days. From each treatment, a 25 g subsample was collected in sterile Whirl-Pak bags (Nasco, Ft. Atkinson, WI) and was mixed in 225 mL of buffered peptone water (Difco Laboratories, Sparks, MD, USA) and stomached for 2 min. The mixtures were serially diluted in 0.1% peptone water and plated on XLD agar (for *Salmonella*), Petrifilm (for STEC) and PDA (for *A. flavus*). The XLD agar, PDA plates and Petrifilms were incubated at 37 °C, 25 °C and 37 °C for 24 h, 72 h and 48 h, respectively, and then colonies were counted. The experiments were replicated three times.

To evaluate the residual antimicrobial effect of the organic acid mixtures on *Salmonella*, uncoated pet food kibbles as described earlier were used. A cocktail culture containing *Salmonella enterica* serovars Enteritidis (ATCC 4931), Heidelberg (ATCC 8326) and Typhimurium (ATCC 14028) was prepared and used as the inoculum (~8 log CFU/mL). The minimum levels of organic acid mixtures Activate DA (1% and 2%) and Activate US WD-MAX at 0.5% and 1%) determined to be effective against *Salmonella* in pet food kibbles were used in this experiment. *Salmonella*-negative kibbles were coated with the minimum effective levels of organic acid mixtures Activate DA (1% and 2%) and Activate US WD-MAX (0.5% and 1%) and divided into one of three challenge groups (for day 1, day 30 and day 90). Organic acid mixture-coated kibble was challenged with *Salmonella* after 1, 30 and 90 days of storage to investigate the residual effect of the organic acid mixtures in the kibble on storage at 25 °C. At each time period, a freshly prepared *Salmonella* inoculum in TSB was spot inoculated on the kibbles using a pipette and mixed thoroughly. The final moisture content of the kibbles post-inoculation was maintained at ~8–9%. After the introduction of the challenge *Salmonella*, the kibble was incubated at 37 °C for 24 h and then analyzed for *Salmonella* counts and enumerated on XLD agar plates. The untreated sample consisted of kibble with no organic acid coating, and the negative control consisted of canola oil-coated kibble (no organic acid and no *Salmonella* inoculation). The experiment was replicated three times.

### 2.5. Confirmative Test for Salmonella

A confirmative test for *Salmonella* for the experiments mentioned above was conducted according to the FDA-BAM method (Bacteriological Analytical Manual). In short, buffered peptone water from the pre-enrichment of each treatment sample, 1.0 mL and 0.1 mL, was transferred to 10 mL of Rappaport-Vassiliadis (RV; BD Difco, Sparks, MD, USA) and tetrathionate (TT; BD Difco, Sparks, MD, USA) broths, respectively, and incubated at 42 °C for 24 h for selective enrichment of *Salmonella*. From each RV and TT broth tubes, one loopful was streaked onto xylose lysine deoxycholate (XLD) agar plates in duplicate. Inverted plates were incubated at 37 °C for 24 h. Presumptive positive *Salmonella* colonies appeared as pink colonies with or without black centers, with the most positive *Salmonella*-producing colonies with large, glossy black centers or almost completely black. Presumptive *Salmonella*-positive colonies from XLD plates were then inoculated into triple sugar iron agar (TSI; BD Difco, Sparks, MD, USA) slants by streaking the slant and stabbing the butt, and lysine iron agar (LIA; BD Difco, Sparks, MD, USA) slants by stabbing the butt twice and then streaking the slant. The TSI and LIA slants were incubated at 37 °C for 24 h. Presumptive *Salmonella*-positive TSI reactions had alkaline (red) slants and acid (yellow) butts, while LIA reactions had alkaline (purple) butts with acidic (yellow) reactions negative for *Salmonella*. All cultures with an alkaline butt in LIA, regardless of TSI reaction, were retained as potential *Salmonella* isolates. Presumed-positive TSI and LIA slant cultures were inoculated into TSB and incubated at 37 °C for 24 h, from which cells were harvested, DNA extracted and confirmed as *Salmonella* based on a molecular analysis [40].

### 2.6. Statistical Analysis

For the challenge study for each of the three pathogens tested (*Salmonella*, STEC and *A. flavus*), the mean log reductions at each sampling time period for the treatments were subjected to a two-way analysis of variance (ANOVA) using the GLIMMIX procedure of statistical software SAS (version 9.3), and the treatment means were separated using Tukey’s post-hoc test when the *F*-test of the ANOVA per treatment was significant at *p <* 0.05 [41]. The linear model *y = a + bx* was fit to a logarithmic reduction in bacterial counts over time (hours) for the treatments, where *a* is the intercept and *b* is the slope. The decimal reduction time or *D*-value (time taken for 1-log reduction of bacterial counts) was calculated as the negative-inverse of slope [42]. 

Similarly, for the determination of the residual antimicrobial effect, the mean log reductions of *Salmonella* at each time period (day 1, day 30, day 90) for the treatments were subjected to a two-way ANOVA, and the treatment means were separated using Tukey’s test (*p <* 0.05) [41].

## 3. Results

The MICs and MBCs of Activate DA against *Salmonella* and STEC ranged from 0.5% to 1% and Activate US WD-MAX ranged from 0.4% to 0.5%. The MIC and MFC of both Activate DA and Activate US WD-MAX against *A. flavus* were 2% (Table 2). Based on the MIC, MBC and MFC results, the treatment concentration levels of Activate DA at 1% and 2% and Activate US WD-MAX at 0.5% and 1% were used subsequently in this study. 

**Figure 2:**
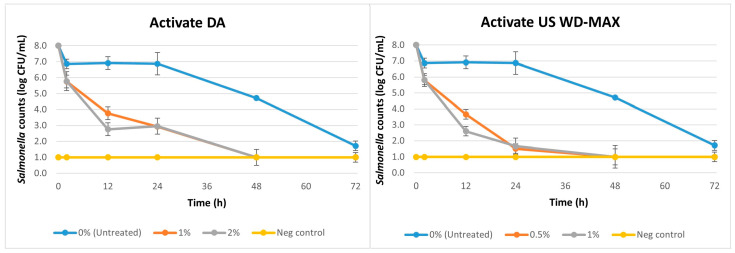
Mean logarithmic counts (log CFU/mL) of *Salmonella* in pet food kibble treatments with inclusion of organic acid mixtures Activate DA at 1% and 2% and Activate US WD-MAX at 0.5% and 1% concentrations as coating in comparison with untreated (0% organic acid). Furthermore, 0 h denotes initial load of *Salmonella* in inoculum before inoculation to the kibble. *Salmonella* counts at day 30 and day 60 are 1 log CFU/mL (not shown). A negative control consisted of canola oil-coated kibble (no organic acid, no inoculation). Limit of detection is 1 log CFU/mL for this study.

For the challenge study against *Salmonella*, the initial load of *Salmonella* in the inoculum was 8 log CFU/mL. A reduction in *Salmonella* counts (*p <* 0.05) was observed over time (2 h to 60 days) due to the inclusion of Activate DA and Activate US WD-MAX as the coating on kibbles as shown in Figure 2 and Table 3. The untreated (no organic acid coating) showed a constant mean *Salmonella* load of 6.9 log CFU/mL until 24 h. By 48 h, the counts decreased to 4.7 log CFU/mL; by 72 h, the counts further reduced to 1.7 log CFU/mL; and by 60 days, the counts declined to 1 log CFU/mL. On an average, a log reduction of 1.1 logs (from an initial load of 8 logs) was observed for the untreated samples until 24 h, 3.3 logs by 48 h, 6.3 logs by 72 h and 7 logs by day 60. The treatments Activate DA and Activate US WD-MAX reduced *Salmonella* counts over time (*p <* 0.05) when compared to the untreated (Table 3). The inclusion of Activate DA at 1% decreased *Salmonella* counts resulting in a population reduction of 5.1 logs (from an initial inoculum load of 8 logs) by 24 h and 7 logs by 48 h. Activate DA at 2% also followed a similar trend decreasing *Salmonella* counts by 5 logs at 24 h and 7 logs at 48 h. However, at 12 h Activate DA at 2% reduced *Salmonella* counts by 5.2 logs whereas at 1% the log reduction was only 4.2 logs at the same time point. Activate US WD-MAX at 0.5% led to a log reduction of 6.5 logs at 24 h, and 7 logs at 48 h. A similar result was observed at 1% with a log reduction of 6.3 logs at 24 h and 7 logs at 48 h. At 12 h, Activate US WD-MAX at 1% reduced *Salmonella* counts by 5.4 logs, whereas at 0.5%, the log reduction was 4.3 logs at the same time point. Overall, there was no difference among the two treatments Activate DA and Activate US WD-MAX across the different concentrations tested (Table 3). Considering the log reductions of *Salmonella* over time, Activate US WD-MAX was effective at a lower concentration (0.5%) than Activate DA. 

**Figure 3:**
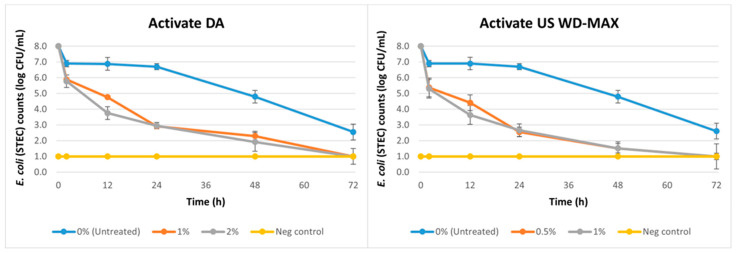
Mean logarithmic counts (log CFU/mL) of *E. coli* (STEC) in pet food kibble treatments with inclusion of organic acid mixtures Activate DA at 1% and 2% and Activate US WD-MAX at 0.5% and 1% concentrations as coating in comparison with untreated (0% organic acid). Furthermore, 0 h denotes initial load of STEC in inoculum before inoculation to the kibble. STEC counts at day 30 and day 60 are 1 log CFU/mL (not shown). A negative control consisted of canola oil-coated kibble (no organic acid, no inoculation). Limit of detection is 1 log CFU/mL for this study.

Similarly, a reduction in STEC counts was observed over time (2 h to 60 days) due to the inclusion of Activate DA and Activate US WD-MAX as a coating on pet food kibbles (Figure 3; Table 4). An average population reduction of 1.1 logs (from an initial load of 8 logs) was observed for the untreated samples until 24 h and then a reduction of 3.2 logs by 48 h, 5.4 logs by 72 h and 7 logs by day 60. The treatments Activate DA and Activate US WD-MAX reduced STEC counts over time (*p <* 0.05) when compared to the untreated (Table 4). The inclusion of Activate DA at 1% decreased STEC counts resulting in a population reduction of 5.1 logs (from an initial load of 8 logs) by 24 h and 5.7 logs by 48 h. Activate DA at 2% also followed a similar trend decreasing STEC counts by 5 logs at 24 h and 6.1 logs at 48 h. However, at 12 h, Activate DA at 2% reduced STEC counts by 4.2 logs, whereas at 1% the population reduction was only 3.2 logs at the same time point. Activate US WD-MAX at 0.5% resulted in a reduction of 5.4 logs at 24 h followed by 6.5 logs at 48 h. At 1%, a similar trend was also observed with a reduction of 5.3 logs at 24 h and 6.5 logs at 48 h. At 12 h, Activate US WD-MAX at 1% reduced STEC counts by 4.4 logs, whereas at 0.5% the log reduction was only 3.6 logs at the same time point. Overall, there was no difference among the two treatments Activate DA and Activate US WD-MAX across the different concentrations tested (Table 4). Considering the log reductions over time, Activate US WD-MAX was effective at a lower dose (0.5%) than the other treatments against STEC. 

**Figure 4:**
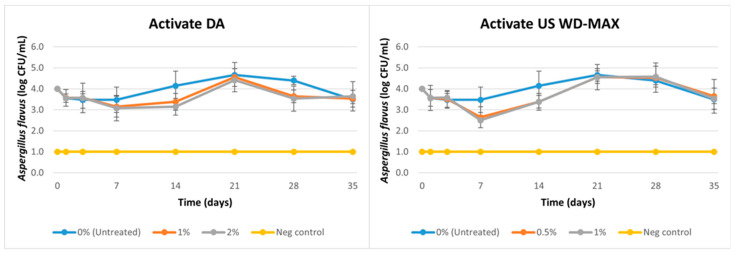
Mean logarithmic counts (log CFU/mL) of *A. flavus* in pet food kibble treatments with inclusion of organic acid mixtures Activate DA at 1% and 2% and Activate US WD-MAX at 0.5% and 1% concentrations as coating in comparison with untreated (0% organic acid). Furthermore, 0 h denotes initial load of *A. flavus* in inoculum before inoculation to the kibble. A negative control consisted of canola oil-coated kibble (no organic acid, no inoculation). Limit of detection is 1 log CFU/mL for this study.

For the challenge study against *A. flavus*, log reductions were not observed consistently over time (1 to 35 days) with the inclusion of Activate DA or Activate US WD-MAX as an oil-based coating on kibbles (Figure 4; Table 5). An average increase of 0.7 logs (from an initial load of 4 logs) was observed for the untreated through day 21 and then a reduction of 0.5 logs by day 35. Overall, the treatments Activate DA and Activate US WD-MAX reduced *A. flavus* counts over time (*p <* 0.05) when compared to the untreated (Table 5). The inclusion of Activate DA at 1% decreased *A. flavus* counts by 0.4-0.9 logs by day 7 and 0.5 logs by day 35. Activate DA at 2% also decreased *A. flavus* counts by 0.9 logs at day 7 and 14, and 0.4 logs at day 35. The inclusion of Activate US WD-MAX at 0.5% resulted in a reduction of 1.4 logs at day 7 followed by an increase of 0.5 logs at day 28 and then a 0.4 log reduction at day 35. At 1%, Activate US WD-MAX showed a reduction of 1.5 logs at day 7 followed by an increase of 0.6 logs at day 28 and then a 0.4 log reduction at day 35. Overall, there was no difference among the two treatments Activate DA and Activate US WD-MAX across the different concentrations tested against *A. flavus* (Table 5). Consistent log reductions of *A. flavus* were not observed over time; however, the increase in mold counts was retarded due to the organic acid treatments compared to the untreated. Activate DA and Activate US WD-MAX exhibited a fungistatic effect during kibble storage but not a fungicidal effect.

The decimal reduction time known as *D*-value is the time (in hours) required to achieve 1-log reduction in bacterial counts. For *Salmonella*, Activate DA had *D*-values ranging from 1.01 to 1.05 h (61–63 min) and Activate US WD-MAX had *D*-values from 1.03 to 1.08 h (62–65 min) (Table 6). However, there was no difference between the two treatments (*p >* 0.05). Similarly, for STEC, Activate DA had *D*-values ranging from 0.98 to 1.02 h (59–62 min) and Activate US WD-MAX had *D*-values from 1.02 to 1.05 h (62–63 min), and there was no difference between the two treatments (*p >* 0.05). *D*-values were not calculated for the effect of organic acid treatments against *A. flavus* as it did not show consistent log reduction of mold counts. Hence, Activate US WD-MAX at the lowest concentration of 0.5% can be considered effective against *Salmonella* and STEC on dry pet food kibbles.

**Figure 5:**
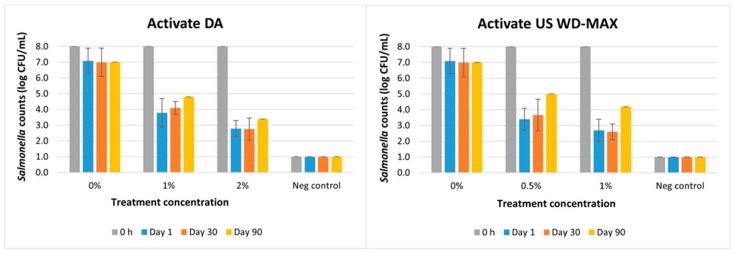
Mean logarithmic counts of *Salmonella* in pet food kibble treatments with inclusion of organic acid mixtures Activate DA at 1% and 2% and Activate US WD-MAX at 0.5% and 1% concentrations as coating, in comparison with untreated (0% organic acid) to investigate residual effect of treatments over 1, 30 and 90 days after repeated exposure to *Salmonella*. A negative control consisted of canola oil-coated kibble (no organic acid, no inoculation). Limit of detection is 1 log CFU/mL for this study.

The untreated and treated (Activate DA and US WD-MAX) kibbles were inoculated with *Salmonella* (~8 log CFU/mL) on day 1, day 30 and day 90 to investigate the residual effect of the treatments over storage time (Figure 5; Table 7). The untreated samples had an initial load of ~7 log CFU/mL of *Salmonella*. Activate US WD-MAX at 0.5% and 1% had a residual antimicrobial effect in the kibbles over time. After inoculation on day 30, analyzing it for *Salmonella* survival resulted in reductions of *Salmonella* by 4.3–5.4 logs. Similarly, Activate DA at 1% and 2% had a residual antimicrobial effect in the kibbles over time (on day 30) and resulted in reductions of *Salmonella* by 3.9–5.2 logs. Inoculating the treated kibbles with *Salmonella* on day 90 resulted in a similar decrease in bacterial counts by 4–5 logs for both Activate DA and Activate US WD-MAX. Compared to the untreated samples, the organic acid treatments reduced *Salmonella* counts (*p <* 0.05); however, there was no difference between the two treatments Activate DA and Activate US WD-MAX (*p >* 0.05) (Table 7). Therefore, both organic acid mixtures can be considered as having a residual antimicrobial effect for 90 days at concentrations 0.5–2% to mitigate the repeated post-processing kibble exposure to *Salmonella*.

## 4. Discussion

The MIC, MBC and MFC tests are in vitro antimicrobial/antifungal susceptibility tests that are usually performed to evaluate the sensitivity of an organism to an antimicrobial or antifungal agent such as an antibiotic or chemical preservative. There have been only a few studies that investigated the antimicrobial properties of organic acid mixtures including HMTBa. Guo-zheng et al. [36] evaluated Activate WD against *Staphylococcus aureus*, *Escherichia coli*, *Salmonella pullorum* and *Campylobacter jejuni* and determined the minimum inhibitory concentration to be 0.3% and minimum bactericidal concentration to be 0.6%. Parker et al. [35] evaluated Activate WD at 0.04% and 0.08% in drinking water for poultry and found a reduction in the horizontal transmission of *Salmonella* in the broiler chickens. These studies show that the organic acid mixtures containing HMTBa have potential antimicrobial effects in feed. From the MIC, MBC and MFC assays in this study, Activate DA (dry formula) and Activate US WD-MAX (wet formula) were both found to be effective against *Salmonella,* STEC and *A. flavus* in nutrient broth (Table 2). From Table 2, Activate US WD-MAX had consistently lower MIC, MBC and MFC results when compared to Activate DA, and hence was slightly more antibacterial against *Salmonella* and STEC and more antimycotic against *A. flavus* than Activate DA. The solubility of an antimicrobial/chemical in the MIC assay plays an important role in reacting with the target organism and its inhibition of growth in the assay. Activate DA is a dry powder which has some solubility issues. Our work was limited by a solubility of 1% in water at 25 °C. There were concerns during the conduct of this research regarding the uniformity in water solution at the various concentrations tested for MIC. It was a powder with a fine particle size after laboratory hammer milling to facilitate this study. The MIC, MBC and MFC of organic acid mixtures against microorganisms depend on several variables including composition and concentration of their components, physical and chemical properties and the culture conditions for the test microorganisms, and thus a comparison of the results with those of other studies involving organic acids is not simple. Iba et al. [43] and Franco et al. [44] reported that the acidification of animal feeds by adding organic acids and organic salts can help control the growth of bacteria and fungi that reduce feed quality and produce toxins. The reduction in bacterial numbers in the animal’s gut and improvement in the balance of gut microflora can potentially have an important prophylactic effect, reducing the opportunity for infection associated with the proliferation of dangerous pathogens in the gut [45,46]. Certain feed additive organic acid mixtures include propionic, formic and butyric acid, which have been used in the past for their antimicrobial properties. These additives have previously been used as a method to control pathogens such as *Salmonella* and *E. coli* in poultry feed and other matrices [47,48]. Of the organic acid mixtures evaluated in this study, Activate DA is a mixture of HMTBa, fumaric acid and benzoic acid, and Activate US WD-MAX is a mixture of HMTBa, lactic acid and phosphoric acid. This combination of acids should have an antimicrobial effect, potentially, as the individual acids in these mixtures were already known to have preservative effects.

It is to be noted that the pH of Activate DA is slightly higher (pH 3) than Activate US WD-MAX (pH 2), and so Activate US WD-MAX has a slightly better acidification property than Activate DA. Activate US WD-MAX, being a liquid formula, is also very miscible in water at all concentrations. Comparing the composition of the mixtures tested, both primarily contain HMTBa, but the secondary constituents differ: fumaric acid and benzoic acid for Activate DA, and lactic acid and phosphoric acid for Activate US WD-MAX. Lactic and phosphoric acids have stronger acidification effects than fumaric acid, which also has solubility issues, and these could be the reasons why Activate US WD-MAX has slightly better antimicrobial properties and hence lower MIC, MBC and MFC than Activate DA. The MICs/MBCs of the organic acid mixtures were similar for *Salmonella* and STEC, while their MIC/MFC against *A. flavus* was higher by 1%. This is because molds are more robust organisms than bacteria, and the *Aspergillus* species was found to be more tolerant to pH and fluctuations in growth conditions [49,50]. So, these molds require greater antimicrobial effects possibly at a higher concentration of the antimicrobial to inhibit their growth. According to Pankey and Sabath [51], when the ratio of MBC to MIC is less than or equal to 4, the antimicrobial can be considered bactericidal. Since the MBC and MFC to MIC ratios of organic acid mixtures in this study were in the range of 1 to 2, they can be considered as antimicrobial and should be effective against *Salmonella,* STEC and *A. flavus* when applied in food systems [52].

The concentrations of antimicrobials tested effectively in a nutrient broth may not be adequate to be tested in a food substrate. This is because several components in the food system such as mineral salts can buffer the effects of antimicrobials. Therefore, it is common practice to test slightly higher concentrations of the antimicrobial in food systems. As the MIC/MBCs of Activate DA were 0.5–1%, we decided to test 1% and 2% of Activate DA for the food substrate kibble challenge study in the challenge studies. Similarly, Activate US WD-MAX had MIC/MBCs of 0.4–0.5% in the broth assay, so we tested it at 0.5% and 1% in kibbles for the challenge studies.

In another study [53] we found that Activate DA at 2% and Activate US WD-MAX at 1% were effective at reducing *Salmonella* counts on food contact surfaces by up to 3.2 logs and 3.5 logs respectively. In this study, for the *Salmonella* and STEC challenge studies, the initial load of bacteria inoculated to the kibbles was ~8 logs. For the untreated samples, while enumerating bacterial loads at the first time point after inoculation, i.e., 2 h, a reduction of about 1 log was observed (Figure 2 and Figure 3). This was due to the limitation in recovery of bacterial loads from the food substrate during analysis. The bacterial loads remained consistent until about 48 h and then started to decline and decreased to about 1 log CFU/mL by 60 days. This was because kibbles are low water activity foods and incubation at 37 °C over an extended period can cause drying of the food substrate which inhibits bacterial growth by desiccation. At 2 h, the organic acid mixtures caused reductions of 2–2.5 logs for *Salmonella* and STEC, and by 12 h, the reductions ranged from 3.6 to 5.4 logs. By 24 h, in the treated kibbles, the organic acid mixtures reduced *Salmonella* loads by 5–6.5 logs and STEC loads by 5–5.4 logs. The organic acid mixture treatments greatly increased the bacterial count reductions within a short time (12 h) compared to the untreated samples. The use of multiple serovars of *Salmonella* and STEC as ‘cocktail inoculums’ in this study further corroborated the strong antimicrobial activity of the two organic acid mixtures.

One might argue that even for the untreated samples there was consistent log reduction in bacterial counts (up to 7 logs) over the 60-day period due to reasons mentioned earlier, and packaged pet food kibbles would normally reach consumers only after several days or weeks. However, we believe that the rapid log reductions of bacterial counts due to the organic acid mixtures help in mitigating cross-contamination during processing and human handling in the processing facility and make the product safer. Also, during changes in storage conditions of the pet food products, such as humid environment, the untreated products may have pathogenic bacterial growth and treatment with these organic acid mixtures can be an effective mitigation strategy.

For the *A. flavus* challenge study, pronounced log reductions of mold counts were not observed in the case of Activate DA and Activate US WD-MAX used as a coating on pet food kibbles (Figure 3). While the untreated sample had a slight increase in mold counts by 0.5 logs by 28 days, the organic acid-treated samples had a slight decrease in mold counts by 1 log by 7 days and then an increase of 0.6 logs in mold counts. As the treated samples differed from the untreated (*p <* 0.05) due to the fact that the increase in mold counts (as seen in the untreated samples) was slightly reduced by the presence of Activate DA and Activate US WD-MAX in the treated samples, we believe the organic acid mixtures had a fungistatic effect on *A. flavus* in the kibbles. Another reason for the static log counts of *A. flavus* over the 35-day period could be because the blank kibbles used in this study already contained 2% vinegar (acetic acid) in their formula (Table 1). Vinegar is typically added as a clean-label preservative ingredient in pet foods for microbial shelf-life stability [54]. It also acts as a mold-inhibiting ingredient, similar to phosphoric acid which is used as a mold inhibitor in semi-moist pet foods. We propose conducting a mold challenge study with Activate DA and Activate US WD-MAX as the coating on kibbles that were manufactured without the inclusion of acids such as vinegar or phosphoric acid to test the effects of these mixtures exclusively on *A. flavus*, with no interference or synergism from other acids. Furthermore, in this challenge study, *A. flavus* counts on the untreated sample did not increase continuously during the 35-day incubation period. There was only a 0.5-log increase by day 35 despite no addition of organic acid mixtures. This was because pet food kibbles are low water activity foods (0.50 a_w_) and incubation at 25 °C for 35 days can result in drying of the food substrate, and hence prevent a mold count increase. Additionally, as it had been mold-challenged with a 4-log inoculum, there can be a competition of nutrients for the growth of mold colonies, which slows their growth.

The *D*-values (Table 6) for *Salmonella* and STEC exposed to Activate DA and Activate US WD-MAX represent the times required for a 10-fold (90% or 1 log) destruction of the initial viable population of the pathogen. The linear regression model *y = a + bx* was best fit for the treatments with higher *R*^2^ values (>0.70) due to pronounced log reductions of the bacterial counts. The *D*-values for *Salmonella* and STEC were about 1 h in this study and there was no difference between the treatments Activate DA and Activate US WD-MAX (*p >* 0.05). Because of the absence of a fungicidal effect or a steady log reduction of mold counts in the case of *A. flavus*, the linear model *y = a + bx* was not fit to the regression over time, and hence *D*-values were not calculated for *A. flavus*.

The residual antimicrobial effect of Activate DA and Activate US WD-MAX coated on pet food kibbles during a storage time from day 1 to day 90 was evaluated (Figure 5), and the treated kibbles when exposed to *Salmonella* on day 30 resulted in a 3.9–5.4 log reduction of the bacteria. On day 90, the *Salmonella* counts were reduced by 3–4.6 logs due to the organic acid treatment’s residual effect during storage. Although not significant, the log reductions on day 90 were slightly lower when compared to day 30, probably because of the buffering effect of other ingredients in the kibble on the organic acids.

The mechanism of the antibacterial activity of organic acids against Gram-negative bacteria such as *Salmonella* has been described in previous research studies [55,56,57,58]. Organic acids in their undissociated and uncharged state are capable of bypassing bacterial cell membranes due to their lipophilic nature. HMTBa has a pKa value of 3.53 (with one active functioning group, i.e., carboxyl group) and being an organic acid remains undissociated in a low pH range (3–5 pH) and thereby able to diffuse into cell membranes of bacteria. Upon entering the more alkaline interior of a bacterium, the anion and proton from organic acids may have deleterious effects on the bacterium by increasing osmotic stress and disrupting important biomolecule synthesis, which finally cause bacterial death. One shortcoming in the method that we realized later in the study was not measuring the water activity after coating the kibbles with the oil plus organic acid mixtures. As it was not a semi-moist pet food product but a dry kibble product with low initial a_w_ (0.50), the coating was performed at a time later than the manufacturing of kibbles that were otherwise stable. We propose measuring water activity following kibble coating in future experiments to better understand if the antimicrobial agents dissolve in these dry products.

While the two organic acid mixtures Activate DA and Activate US WD-MAX showed promising antibacterial properties against *Salmonella* and STEC when applied as a coating on pet food kibbles and have a strong residual antimicrobial effect over extended storage times on the kibbles, their acceptability to pets needs to be evaluated. Even though these have been in use as supplements in animal feed, we speculate that coating them on kibbles may have a strong effect of smell/flavor to the pets, which may lead to differences in acceptability. Dhakal and Aldrich [6] reported that coating dog food kibbles with a combination of medium chain fatty acids (caproic, caprylic and capric) was effective in mitigating *Salmonella* but reduced the acceptability of the kibbles due to strong aroma of the organic acids. Therefore, for this study, we propose conducting palatability tests, such as a two-bowl forced choice evaluation test [6], to determine the acceptability of these organic acid-coated kibbles by pets. In case of any changes in acceptability of these kibbles, a palatant may be necessary as a further coating to mask the aroma or flavor of the organic acid mixtures when applied to kibbles. Recently, combinations of organic acids and medium chain fatty acids (e.g., lauric acid) have also demonstrated synergistic benefits on animal intestinal health due to their antibacterial properties [59]. For future work, we propose investigating the synergistic effects of HMTBa with medium chain fatty acids as coating on pet food kibbles to control pathogen recontamination. Furthermore, it is more likely for pathogens such as *Salmonella* to be reintroduced to kibbles post-processing via dust, flies or employee-handling as opposed to a liquid contamination source. Therefore, we propose conducting challenge studies using a dry inoculum of *Salmonella* as water-based inoculum can have aversion to the oil-based surface coating on the kibbles. Additionally, it would be interesting to measure the production of aflatoxins in future challenge studies with *A. flavus* to study whether the mold is more sensitive to organic acids inhibiting mycotoxin production than growth.

## 5. Conclusions

The results of this study indicate that the use of organic acid mixtures containing HMTBa, Activate DA and Activate US WD-MAX, as a coating ingredient on dry pet food kibbles showed a promising effect as a food-safe ingredient to mitigate post-processing *Salmonella* and STEC contamination. Being effective at a low concentration among the treatments tested and for ease of application with no solubility issues, we believe that Activate US WD-MAX at 0.5% or 1% was the most effective to be used as a kibble coating to control *Salmonella* and STEC contamination.

## Figures and Tables

**Table 1:** Formulation of uncoated dry pet food kibbles.

Ingredient	Percent *w*/*w*
Chicken meal	42.6
Corn meal	17.7
Wheat flour	17.7
Rice flour	17.7
Vinegar	2.0
Salt	0.5
Potassium chloride	0.3
Choline chloride	0.3
Dicalcium phosphate	0.3
Calcium carbonate	0.3
Trace mineral premix	0.2
Vitamin premix	0.1
Fish oil	0.1
Taurine	0.1
Natural antioxidant	0.1
Total	100.0

**Table 2:** Minimum inhibitory concentrations (MICs), minimum bactericidal concentrations (MBCs) and minimum fungicidal concentrations (MFCs) of organic acid mixtures Activate DA and Activate US WD-MAX in nutrient broth (TSB or PDB) against *Salmonella, Escherichia coli* (STEC) and *Aspergillus flavus*. A positive control consisted of bacterial or fungal inoculum only (no treatment), and a negative control consisted of tryptic soy broth (TSB) or potato dextrose broth (PDB) only (no treatment, no inoculation).

Treatment	*Salmonella*		*E. coli* (STEC)		*A. flavus*	
	MIC ^1^ (%)	MBC ^1^ (%)	MIC ^1^ (%)	MBC ^1^ (%)	MIC ^1^ (%)	MFC ^1^ (%)
Activate DA	0.5	1.0	1.0	1.0	2.0	2.0
Activate US WD-MAX	0.4	0.5	0.4	0.5	1.0	1.0

^1^ The most recurring value of three replicates is reported as MIC, MBC or MFC.

**Table 3:** Mean logarithmic reduction of *Salmonella* counts in pet food kibble treatments with inclusion of organic acid mixtures Activate DA at 1% and 2% and Activate US WD-MAX at 0.5% and 1% concentrations as coating, in comparison with untreated (0% organic acid). The logarithmic reductions were calculated as the difference between *Salmonella* counts in the inoculum (8 log CFU/mL) and on the kibbles (untreated and treated). A negative control consisted of canola oil-coated kibble (no organic acid, no inoculation).

Time	*Salmonella* Log Reduction (log CFU/mL) (Mean ± SE) ^1,2,3^				
	Untreated	Activate US WD-MAX		Activate DA	
	0.0%	0.5%	1.0%	1.0%	2.0%
0 h	0.0 ± 0.0 ^a,A^	0.0 ± 0.0 ^b,A^	0.0 ± 0.0 ^b,A^	0.0 ± 0.0 ^b,A^	0.0 ± 0.0 ^b,A^
2 h	1.1 ± 0.3 ^a,B^	2.2 ± 0.3 ^b,B^	2.2 ± 0.4 ^b,B^	2.2 ± 0.4 ^b,B^	2.2 ± 0.6 ^b,B^
12 h	1.1 ± 0.4 ^a,C^	4.3 ± 0.3 ^b,C^	5.4 ± 0.3 ^b,C^	4.2 ± 0.4 ^b,C^	5.2 ± 0.4 ^b,C^
24 h	1.1 ± 0.7 ^a,C^	6.5 ± 0.3 ^b,C^	6.3 ± 0.5 ^b,C^	5.1 ± 0.1 ^b,C^	5.0 ± 0.5 ^b,C^
48 h	3.3 ± 0.0 ^a,D^	7.0 ± 0.7 ^b,D^	7.0 ± 0.5 ^b,D^	7.0 ± 0.5 ^b,D^	7.0 ± 0.1 ^b,D^
72 h	6.3 ± 0.3 ^a,D^	7.0 ± 0.3 ^b,D^	7.0 ± 0.1 ^b,D^	7.0 ± 0.3 ^b,D^	7.0 ± 0.3 ^b,D^
30 d	7.0 ± 0.5 ^a,D^	7.0 ± 0.1 ^b,D^	7.0 ± 0.7 ^b,D^	7.0 ± 0.6 ^b,D^	7.0 ± 0.4 ^b,D^
60 d	7.0 ± 0.5 ^a,D^	7.0 ± 0.4 ^b,D^	7.0 ± 0.6 ^b,D^	7.0 ± 0.7 ^b,D^	7.0 ± 0.6 ^b,D^

^1^ Each mean is based on *n* = 3 replications. ^2^ Means among the treatments across concentrations followed by different letters in lower case are significantly different (*p <* 0.05, Tukey’s test). ^3^ Within each treatment, means among different times followed by different letters in upper case are significantly different (*p <* 0.05, Tukey’s test).

**Table 4:** Mean logarithmic reduction of *E. coli* (STEC) counts in pet food kibble treatments with inclusion of organic acid mixtures Activate DA at 1% and 2% and Activate US WD-MAX at 0.5% and 1% concentrations as coating, in comparison with untreated (0% organic acid). The logarithmic reductions were calculated as the difference between *E. coli* counts in the inoculum (8 log CFU/mL) and on the kibbles (untreated and treated). A negative control consisted of canola oil-coated kibble (no organic acid, no inoculation).

Time	*E. coli* (STEC) Log Reduction (log CFU/mL) (Mean ± SE) ^1,2,3^				
	Untreated	Activate US WD-MAX		Activate DA	
	0.0%	0.5%	1.0%	1.0%	2.0%
0 h	0.0 ± 0.0 ^a,A^	0.0 ± 0.0 ^b,A^	0.0 ± 0.0 ^b,A^	0.0 ± 0.0 ^b,A^	0.0 ± 0.0 ^b,A^
2 h	1.1 ± 0.2 ^a,B^	2.6 ± 0.6 ^b,B^	2.7 ± 0.6 ^b,B^	2.1 ± 0.1 ^b,B^	2.2 ± 0.4 ^b,B^
12 h	1.1 ± 0.4 ^a,C^	3.6 ± 0.5 ^b,C^	4.4 ± 0.6 ^b,C^	3.2 ± 0.1 ^b,C^	4.2 ± 0.4 ^b,C^
24 h	1.3 ± 0.2 ^a,D^	5.4 ± 0.3 ^b,D^	5.3 ± 0.4 ^b,D^	5.1 ± 0.1 ^b,D^	5.0 ± 0.2 ^b,D^
48 h	3.2 ± 0.4 ^a,E^	6.5 ± 0.3 ^b,E^	6.5 ± 0.4 ^b,E^	5.7 ± 0.3 ^b,E^	6.1 ± 0.6 ^b,E^
72 h	5.4 ± 0.5 ^a,F^	7.0 ± 0.8 ^b,F^	7.0 ± 0.2 ^b,F^	7.0 ± 0.1 ^b,F^	7.0 ± 0.5 ^b,F^
30 d	7.0 ± 0.5 ^a,F^	7.0 ± 0.3 ^b,F^	7.0 ± 0.5 ^b,F^	7.0 ± 0.2 ^b,F^	7.0 ± 0.4 ^b,F^
60 d	7.0 ± 0.2 ^a,F^	7.0 ± 0.2 ^b,F^	7.0 ± 0.7 ^b,F^	7.0 ± 0.4 ^b,F^	7.0 ± 0.7 ^b,F^

^1^ Each mean is based on *n* = 3 replications. ^2^ Means among the treatments across concentrations followed by different letters in lower case are significantly different (*p <* 0.05, Tukey’s test). ^3^ Within each treatment, means among different times followed by different letters in upper case are significantly different (*p <* 0.05, Tukey’s test).

**Table 5:** Mean logarithmic reduction of *Aspergillus flavus* counts in pet food kibble treatments with inclusion of organic acid mixtures Activate DA at 1% and 2% and Activate US WD-MAX at 0.5% and 1% concentrations as coating, in comparison with untreated (0% organic acid). The logarithmic reductions were calculated as the difference between *A. flavus* counts in the inoculum (4 log CFU/mL) and on the kibbles (untreated and treated). A negative control consisted of canola oil-coated kibble (no organic acid, no inoculation).

Time	*A. flavus* Log Reduction (log CFU/mL) (Mean ± SE) ^1,2,3^				
	Untreated	Activate US WD-MAX		Activate DA	
	0.0%	0.5%	1.0%	1.0%	2.0%
0 h	0.0 ± 0.0 ^a,B^	0.0 ± 0.0 ^b,B^	0.0 ± 0.0 ^b,B^	0.0 ± 0.0 ^b,B^	0.0 ± 0.0 ^b,B^
1 d	0.4 ± 0.1 ^a,C^	0.4 ± 0.6 ^b,C^	0.4 ± 0.4 ^b,C^	0.4 ± 0.4 ^b,C^	0.4 ± 0.2 ^b,C^
3 d	0.5 ± 0.4 ^a,C^	0.5 ± 0.4 ^b,C^	0.4 ± 0.2 ^b,C^	0.4 ± 0.2 ^b,C^	0.4 ± 0.7 ^b,C^
7 d	0.5 ± 0.6 ^a,D^	1.4 ± 0.5 ^b,D^	1.5 ± 0.1 ^b,D^	0.9 ± 0.5 ^b,D^	0.9 ± 0.6 ^b,D^
14 d	−0.1 ± 0.7 ^a,C^	0.6 ± 0.4 ^b,C^	0.6 ± 0.3 ^b,C^	0.6 ± 0.4 ^b,C^	0.9 ± 0.4 ^b,C^
21 d	−0.7 ± 0.3 ^a,A^	−0.6 ± 0.6 ^b,A^	−0.6 ± 0.3 ^b,A^	−0.6 ± 0.7 ^b,A^	−0.4 ± 0.3 ^b,A^
28 d	−0.4 ± 0.2 ^a,B^	−0.5 ± 0.7 ^b,B^	−0.6 ± 0.5 ^b,B^	0.4 ± 0.3 ^b,B^	0.5 ± 0.6 ^b,B^
35 d	0.5 ± 0.2 ^a,C^	0.4 ± 0.8 ^b,C^	0.5 ± 0.5 ^b,C^	0.5 ± 0.4 ^b,C^	0.4 ± 0.7 ^b,C^

^1^ Each mean is based on *n* = 3 replications. ^2^ Means among the treatments across concentrations followed by different letters in lower case are significantly different (*p <* 0.05, Tukey’s test). ^3^ Within each treatment, means among different times followed by different letters in upper case are significantly different (*p <* 0.05, Tukey’s test).

**Table 6:** Linear regression (linear model *y = a + bx*) parameters of logarithmic reduction of *Salmonella* and *E. coli* (STEC) counts in pet food kibble treatments with inclusion of organic acid mixtures Activate DA at 1% and 2% and Activate US WD-MAX at 0.5% and 1% concentrations as coating.

Treatment	Concentration (%)	Linear Regression Parameters			
		*a*	*b*	*R* ^2^	*D*-Value ^1,2^ (h)
*Salmonella*:					
Activate US WD-MAX	0.5	7.24	−0.97	0.77	1.03
	1.0	6.97	−0.93	0.73	1.08
Activate DA	1.0	7.50	−0.99	0.83	1.01
	2.0	7.22	−0.95	0.78	1.05
*E. coli* (STEC):					
Activate US WD-MAX	0.5	7.51	−0.98	0.86	1.02
	1.0	7.28	−0.95	0.84	1.05
Activate DA	1.0	7.93	−1.02	0.91	0.98
	2.0	7.58	−0.98	0.87	1.02

*a* and *b* are linear regression parameters; *a* = intercept; *b* = slope. ^1^ *D*-value (−1/*b*) shows the decimal reduction time (in hours) for 1-log reduction of *Salmonella* and *E. coli* counts. ^2^ *D*-values were not calculated for treatments showing no log reduction of *A. flavus* counts over time.

**Table 7:** Mean logarithmic reduction of *Salmonella* counts in pet food kibble treatments with inclusion of organic acid mixtures Activate DA at 1% and 2% and Activate US WD-MAX at 0.5% and 1% concentrations as coating, in comparison with untreated (0% organic acid), showing residual effect of treatments over 1, 30 and 90 days during storage. The logarithmic reductions were calculated as the difference between *Salmonella* counts in the inoculum (8 log CFU/mL) and on the kibbles (untreated and treated). A negative control consisted of canola oil-coated kibble (no organic acid, no inoculation).

Time	*Salmonella* Log Reduction (log CFU/mL) (Mean ± SE) ^1,2,3^				
	Untreated	Activate US WD-MAX		Activate DA	
	0.0%	0.5%	1.0%	1.0%	2.0%
0 h	0.0 ± 0.0 ^a,A^	0.0 ± 0.0 ^b,A^	0.0 ± 0.0 ^b,A^	0.0 ± 0.0 ^b,A^	0.0 ± 0.0 ^b,A^
1 d	0.9 ± 0.8 ^a,B^	4.6 ± 0.7 ^b,B^	5.3 ± 0.7 ^b,B^	4.2 ± 0.9 ^b,B^	5.2 ± 0.5 ^b,B^
30 d	1.0 ± 0.9 ^a,B^	4.3 ± 1.0 ^b,B^	5.4 ± 0.5 ^b,B^	3.9 ± 0.4 ^b,B^	5.2 ± 0.7 ^b,B^
90 d	1.0 ± 0.8 ^a,B^	3.0 ± 0.6 ^b,B^	3.8 ± 0.4 ^b,B^	3.2 ± 0.7 ^b,B^	4.6 ± 0.4 ^b,B^

^1^ Each mean is based on *n* = 3 replications. ^2^ Means among the treatments across concentrations followed by different letters in lower case are significantly different (*p <* 0.05, Tukey’s test). ^3^ Within each treatment, means among different times followed by different letters in upper case are significantly different (*p <* 0.05, Tukey’s test).

## Data Availability

Data will be provided upon reasonable request by author Aiswariya Deliephan.
